# Supplementary material for: Active learning-assisted neutron spectroscopy with log-Gaussian processes
Source: Nat Commun. 2023 Apr 19;14:2246. doi: 10.1038/s41467-023-37418-8 (PMC10115805; doi:10.1038/s41467-023-37418-8)
Supplement: Supplementary file 1 — Supplementary Information [file 41467_2023_37418_MOESM1_ESM.pdf]

Active learning-assisted neutron spectroscopy with log-Gaussian processes

—

Supplementary Information

M. Teixeira Parente[1,†], G. Brandl[1], C. Franz[1], U. Stuhr[2],
M. Ganeva[1,‡] and A. Schneidewind[1]

[1]Jülich Centre for Neutron Science (JCNS) at Heinz Maier-Leibnitz Zentrum (MLZ),
Forschungszentrum Jülich, Garching, Germany

[2]Laboratory for Neutron Scattering and Imaging, Paul Scherrer Institute (PSI), Villigen, Switzerland

Contents

Supplementary Note 1 **2**

Supplementary Note 2 **4**

Supplementary Note 3 **5**

Supplementary Note 4 **6**

Supplementary Note 5 **7**

Supplementary Note 6 **8**

Supplementary Figures **9**

Supplementary References **21**

[†]Corresponding author 1: Lichtenbergstraße 1, 85748 Garching, Germany, m.teixeira.parente@fz-juelich.de.
[‡]Corresponding author 2: Wilhelm-Johnen-Straße, 52428 Jülich, Germany, m.ganeva@fz-juelich.de.

Supplementary Note 1 TAS setting

In TAS experiments, intensities are observed at a certain point in the $\mathbf{Q}$-E space of the investigated material by counting scattered neutrons on a detector device. The momentum space $\mathbf{Q}$ is the Fourier transform of an initial periodic spatial lattice depending on the material and provides wavevector coordinates $\mathbf{q} = (h, k, l)^\top \in \mathbf{Q}$ (Miller indices) in relative lattice units (r.l.u.) to move within it. The one-dimensional energy space E provides a coordinate $\omega \in E$, mostly measured in units of milli-electron volts (meV), to describe energy transfer. Neglecting units, $\mathbf{Q}$-E space can be thought of as a four-dimensional real space, i. e., $(\mathbf{q}, \omega)^\top \in \mathbf{R}^r$, $r = 4$.

TAS experiments are, however, often not carried out in full-dimensional $\mathbf{Q}$-E space but on a lower-dimensional (often two-dimensional) hyperplane. The coordinates on the corresponding hyperplane in $\mathbf{Q}$-E space are denoted by $\mathbf{x} = (x_1, \ldots, x_n)^\top \in \mathbf{R}^n$, $n \leq r$, and the transformation $T : \mathbf{R}^n \to \mathbf{R}^r$ is defined as

$$\begin{pmatrix} \mathbf{q} \\ \omega \end{pmatrix} = W\mathbf{x} + \mathbf{b} =: T(\mathbf{x}) \tag{1}$$

for a full-rank transformation matrix $W \in \mathbf{R}^{r \times n}$ and an offset $\mathbf{b} \in \mathbf{R}^r$. Most often, the transformation matrix has the shape

$$W = \begin{pmatrix} * & 0 \\ * & 0 \\ * & 0 \\ 0 & 1 \end{pmatrix} \tag{2}$$

meaning that a two-dimensional hyperplane is spanned by a certain direction in $\mathbf{Q}$ space and energy transfer. The re-transformation $T^{-1} : \mathbf{R}^r \to \mathbf{R}^n$, an orthogonal projection onto the hyperplane, is then given by

$$\mathbf{x} = (W^\top W)^{-1} W^\top \left[\begin{pmatrix} \mathbf{q} \\ \omega \end{pmatrix} - \mathbf{b} \right] =: T^{-1}(\mathbf{q}, \omega). \tag{3}$$

Note that $T^{-1}(T(\mathbf{x})) = \mathbf{x}$ for each $\mathbf{x} \in \mathbf{R}^n$, but $T(T^{-1}(\mathbf{q}, \omega)) = (\mathbf{q}, \omega)^\top$ only for $(\mathbf{q}, \omega)^\top \in T(\mathbf{R}^n)$.

The rectangular set $\mathcal{X} \subseteq \mathbf{R}^n$, becoming the domain of the intensity function, is defined as

$$\mathcal{X} \coloneqq [x_1^-, x_1^+] \times \cdots \times [x_n^-, x_n^+] \tag{4}$$

for given limits of investigation $x_k^\pm \in \mathbf{R}$, $k \in \{1, \ldots, n\}$. For example, in Fig. 1, we have $(x_1^-, x_1^+, x_2^-, x_2^+) = (2.3, 3.3, 2.5, 5.5)$.

In order to observe intensities at a certain location in $\mathbf{Q}$-E space, a TAS needs to move its main axes to corresponding angles. In the constant-k_f mode we used for our experimental setups, it is enough to regard a subset of three angles and their angular velocities $\mathbf{v} = (v_1, v_2, v_3)^\top \in [0, \infty)^3$. For the connection between points in $\mathbf{Q}$-E space and their respective angles of the instrument axes, we formally use an angle map

$$\mathbf{\Psi} : \mathrm{dom}(\mathbf{\Psi}) \to [0, \pi)^3, \ (\mathbf{q}, e) \mapsto (\Psi_1(\mathbf{q}, e), \Psi_2(\mathbf{q}, e), \Psi_3(\mathbf{q}, e))^\top \tag{5}$$

with domain $\mathrm{dom}(\mathbf{\Psi}) \subseteq \mathbf{R}^r$ containing points in $\mathbf{Q}$-E space for which $\mathbf{\Psi}$ is well-defined, i. e., points reachable by the instrument. Translating this domain to the lower-dimensional coordinates, we define

$$\mathcal{X}^* \coloneqq T^{-1}(\mathrm{dom}(\mathbf{\Psi}) \cap T(\mathcal{X})) \tag{6}$$

as the set of points $\mathbf{x} \in \mathcal{X}$ such that, by abuse of notation, the map

$$\mathbf{\Psi}(\mathbf{x}) \coloneqq \mathbf{\Psi}(T(\mathbf{x})) \tag{7}$$

is well-defined. We assume that the limits of investigation are set such that $\mathbf{\Psi} : \mathcal{X}^* \to [0, \pi)^3$ becomes an injective function.

The sample and its orientation induce a scattering function $s : T(\mathcal{X}) \to [0, \infty)$ describing intensities theoretically present. For $\mathbf{x} \in \mathcal{X}$, we define again

$$s(\mathbf{x}) \coloneqq s(T(\mathbf{x})). \tag{8}$$

The scattering function is not directly accessible due to limits in the instrument resolution. If we denote the resolution function by $\varphi : T(\mathcal{X}^*) \to (T(\mathcal{X}^*) \to [0, \infty))$ and again define

$$\varphi(\mathbf{x}) \coloneqq \varphi(T(\mathbf{x})) \tag{9}$$

for $\mathbf{x} \in \mathcal{X}^*$, we eventually get the intensity function $i : \mathcal{X}^* \to [0, \infty)$ as the convolution of s with φ, i. e.,

$$i(\mathbf{x}) \coloneqq (s * \varphi(\mathbf{x}))(\mathbf{x}). \tag{10}$$

Finally, the cost for moving the instrument axes, i. e., changing their angles, from a certain location $\mathbf{x} \in \mathcal{X}^*$ to another $\mathbf{x}' \in \mathcal{X}^*$ is formalized by the metric $d : \mathcal{X}^* \times \mathcal{X}^* \to [0, \infty)$ defined as

$$d(\mathbf{x}, \mathbf{x}') \coloneqq \max_{k \in \{1,2,3\}} \left| \frac{\Psi_k(\mathbf{x}) - \Psi_k(\mathbf{x}')}{v_k} \right|, \tag{11}$$

where Ψ_k are components of the angle map from Eq. (5) and v_k denote the corresponding angular velocities of the instrument. It indicates the maximum time needed changing all angles in parallel. Note that d is indeed a metric in the mathematical sense since the angle map is chosen to be injective.

Supplementary Note 2 Setup for neutron experiment

To prepare the workflow for a neutron experiment at a TAS, we tested real space movements of instrument axes as well as the communication between the software implementation of our approach and the instrument control system NICOS during dry runs, i. e., without neutrons, at the cold TAS PANDA (MLZ) [1] for several excitations. Having a lack of neutron beam time in Europe currently, we are grateful for the granted beam time at the thermal TAS EIGER (PSI) [2] making a real experiment possible to finally demonstrate the usefulness and benefits of our approach.

The experimental setup at EIGER was as follows. We oriented a SnTe sample (space group 225) in the (hhl) scattering plane and mounted it in a closed cycle cryostat for background decrease, even though we have been measuring at room temperature. EIGER was operated in constant-k_f mode ($k_f = 2.66$ Å^{-1}) with a PG filter on k_f, a double-focusing PG002 monochromator, and a horizontally focusing PG002 analyzer. In scenarios 1 and 2, we counted neutrons on the detector device at each measurement location in $\mathbf{Q}$-E space until $100,000$ neutrons were counted on the monitor device, whereas in scenario 3, we counted for $40,000$ (initialization) and $50,000$ (after initialization) monitor counts, respectively.

For scenarios 1 and 2, due to the coupling of LA and TO, with an additional TA mode, the intensity distribution provides an ideal setting including real background, strong and weak signals, and symmetry breaking in intensity along the $\mathbf{Q}$ direction.

Supplementary Note 3 Milestone values for benchmark

The milestone values used for the benchmark are given in Supplementary Table 1. Recall that, for each test case, they are determined by the four stages (I-IV) of the grid approach (Supplementary Fig. 2). The intensity functions for all test cases are displayed in Supplementary Fig. 3 a)-t).

Test case	Milestone values in hours			
	I	II	III	IV
a)	2.28	4.28	6.27	8.02
b)	2.27	4.27	6.25	8.00
c)	2.23	4.45	6.66	8.90
d)	2.37	4.46	6.53	8.34
e)	2.17	4.27	6.42	8.57
f)	2.21	4.41	6.61	8.82
g)	2.18	4.37	6.55	8.73
h)	2.19	4.38	6.57	8.76
i)	2.21	4.42	6.63	8.84
j)	2.17	4.34	6.50	8.67
k)	2.18	4.36	6.53	8.71
l)	2.21	4.43	6.64	8.85
m)	2.16	4.32	6.48	8.64
n)	2.23	4.47	6.70	8.93
o)	2.17	4.34	6.51	8.69
p)	2.15	4.30	6.45	8.60
q)	2.15	4.30	6.45	8.60
r)	1.80	3.58	5.36	7.14
s)	1.80	3.34	4.87	6.18
t)	1.74	3.47	5.20	6.93

Supplementary Table 1: Milestone values for each benchmark test case.

Supplementary Note 4 Comparison with gpCAM

We emphasize that the following comparison was made based on all the information available to us about gpCAM and its correct use. Since gpCAM, as mentioned in the Results section, does not specify how to choose the acquisition function in the TAS setting, we use the same as that used in a former neutron experiment at ThALES [3, Eq. (6)], i. e.,

$$\mathrm{acq}_J(\mathbf{x}) = \tilde{\sigma}_J(\mathbf{x}) + 3\,\tilde{m}_J(\mathbf{x})\,\tilde{\sigma}_J(\mathbf{x}), \tag{12}$$

where $\tilde{m}_J$ and $\tilde{\sigma}_J$ denote the posterior mean and, respectively, standard deviation function of the Gaussian process that gpCAM fits to intensity observations directly, i. e., to original data without any transformation. Note that we only used this one acquisition function (Eq. (12)). Other acquisition functions may yield fundamentally different results.

Our implementation using version 7.4.4 of gpCAM can be accessed at the repository `jugit.fz-juelich.de/ainx/base-fork-ariane` (branch: `gpcam-test`, commit SHA: `f9acd9a6`, file: `tas/approaches/_gpcam.py`).

As with our approach, we ran gpCAM for each benchmark test case in different variants and performed 100 repetitions with different random seeds for each to see the variability of their results caused by stochastic components. The variants, differing only in the way of initializing the Gaussian process and considering the cost function c_J (Eq. (3) in the main text), were:

1. initialization uniformly at random and considering c_J (default),
2. initialization uniformly at random and ignoring c_J,
3. initialization with the grid used for our approach (Supplementary Fig. 7) and considering c_J.

Examples of particular experiments performed by gpCAM in the default setting (variant 1) for each test case are depicted in Supplementary Fig. 9. We see that the quality of experiments for most spin wave test cases (Supplementary Fig. 9f-q) is rather poor. In fact, it can be observed that gpCAM places measurement points near the edges and very close to each other after initialization and thus does not detect regions of signal at all in these test cases. There are, however, some test cases where gpCAM performs as expected to some extent, but all of them share the commonality of having relatively large regions of signal. Interestingly, the target intensity function underlying Supplementary Fig. 9d (Supplementary Fig. 3d), for which gpCAM performed reasonably well but failed to detect the region of weak signal, bears a fairly strong resemblance to the intensity function from the neutron experiment at ThALES [3, Fig. 4e]. Note that also the other two variants (variants 2 and 3) perform experiments of a similar poor quality in this benchmark.

Not surprisingly, the poor quality of gpCAM's particular experiments displayed in Supplementary Fig. 9 is also seen in the quantitative benchmark results, which we provide in Supplementary Fig. 10, analogous to Fig. 6, for the sake of completeness. We observe that our approach performs substantially better in each test case than all gpCAM variants. In particular, the results of variants with random initialization (variants 1 and 2) show not only a poor median average but also a large variability. The variant with the same initialization grid as our approach (variant 3) performs better than the other two, but this is only due to the fact that the initialization grid already covers the whole domain of interest $\mathcal{X}$. In fact, this variant does not significantly improve the benefit of its experiments after initialization, especially in the spin wave test cases mentioned above.

Supplementary Note 5 Further results from neutron experiment

We have additionally tested our approach in the setting of scenario 1 with different values for the background level and intensity threshold. The results (Supplementary Fig. 11) further support the claim that our approach identifies regions of signal successfully and that a change in the intensity threshold (from $\tau_3 = 90$ to $\tau_4 = 130$, both with $\gamma_3 = \gamma_4 = 45$) does not have significant influence on the final outcome. On the contrary, we can see again that the particular value of the intensity threshold influences the width of the branches the measurements are placed on, which additionally substantiates our claim of interpretability and explainability (see Discussion section).

In scenario 3, we have investigated the material SnTe on $\mathcal{X} = [0,1] \times [1,12]$ along the $\mathbf{Q}$ direction $(1,1,0)$ again but with offset $(0,0,2)$ (instead of $(0,0,3)$) and energy transfer. As the corresponding intensity distribution in this setting has been unknown to us, we have had a scenario that required searching for signals of interest and thus a typical situation for the productive application of our approach in the future. Note that we used our approach in the default setting (Table 1) for this scenario, i. e., with an automated estimation of the background level ($\gamma = 12$) and the intensity threshold ($\tau = 54$). The results (Supplementary Fig. 12) demonstrate again that, after initialization, a large part of measurement points is placed in the only region of signal. In particular, although the signals vary greatly in magnitude, the measurement points are evenly distributed in this region, which is due to a well-estimated intensity threshold. The evolution of the experiment related to this scenario can be seen in Supplementary Movie 5.

Supplementary Note 6 Proof of Proposition 1

Lemma 1. For each $y \in \mathbf{R}$, it holds that

$$\lim_{\sigma \to 0^+} \frac{\exp(\sigma y) - \exp(\sigma^2/2)}{(\exp(\sigma^2) - 1)^{1/2} \cdot \exp(\sigma^2/2)} = y. \tag{13}$$

Proof. Let $y \in \mathbf{R}$. Note that

$$\lim_{x \to 0} \frac{\exp(x) - 1}{x} = 1 \tag{14}$$

using the Taylor expansion of $\exp(x)$. With this in mind, we compute

$$\frac{\exp(\sigma y) - \exp(\sigma^2/2)}{(\exp(\sigma^2) - 1)^{1/2} \cdot \exp(\sigma^2/2)} = \frac{\frac{\exp(\sigma y)-1}{\sigma y} \cdot \sigma y - \frac{\exp(\sigma^2/2)-1}{\sigma^2/2} \cdot \sigma^2/2}{\left(\frac{\exp(\sigma^2)-1}{\sigma^2}\right)^{1/2} \cdot \sigma \cdot \exp(\sigma^2/2)}. \tag{15}$$

Cancelling σ from both, the numerator and the denominator, gives

$$\begin{aligned}
\lim_{\sigma \to 0^+} \frac{\exp(\sigma y) - \exp(\sigma^2/2)}{(\exp(\sigma^2) - 1)^{1/2} \cdot \exp(\sigma^2/2)} &= \lim_{\sigma \to 0^+} \frac{\frac{\exp(\sigma y)-1}{\sigma y} \cdot y - \frac{\exp(\sigma^2/2)-1}{\sigma^2/2} \cdot \sigma/2}{\left(\frac{\exp(\sigma^2)-1}{\sigma^2}\right)^{1/2} \cdot \exp(\sigma^2/2)} \\
&= \frac{1 \cdot y - 1 \cdot 0}{1 \cdot 1} \\
&= y.
\end{aligned} \tag{16}$$

Proof of Proposition 1. First, we compute

$$\begin{aligned}
\frac{Z}{\sqrt{\mathbf{V}\mathrm{ar}(Z)}} &= \exp\left(\log\left(\frac{1}{\sqrt{\mathbf{V}\mathrm{ar}(Z)}}\right) + \mu + \sigma\eta\right) && (17)\\
&= \exp\left(-\frac{1}{2}\log(\mathbf{V}\mathrm{ar}(Z)) + \mu + \sigma\eta\right) && (18)\\
&= \exp\left(-\frac{1}{2}[\log(\exp(\sigma^2) - 1) + 2\mu + \sigma^2] + \mu + \sigma\eta\right) && (19)\\
&= \exp\left(-\frac{1}{2}\log(\exp(\sigma^2) - 1) - \frac{\sigma^2}{2} + \sigma\eta\right) && (20)\\
&= \frac{1}{(\exp(\sigma^2) - 1)^{1/2}} \cdot \exp\left(-\frac{\sigma^2}{2} + \sigma\eta\right) && (21)\\
&= \frac{\exp(\sigma\eta)}{(\exp(\sigma^2) - 1)^{1/2} \cdot \exp(\sigma^2/2)} && (22)
\end{aligned}$$

and

$$\begin{aligned}
\frac{\mathbf{E}[Z]}{\sqrt{\mathbf{V}\mathrm{ar}(Z)}} &= \frac{\exp\left(\mu + \frac{\sigma^2}{2}\right)}{(\exp(\sigma^2) - 1)^{1/2} \cdot \exp(\mu + \sigma^2/2)} && (23)\\
&= \frac{\exp(\sigma^2/2)}{(\exp(\sigma^2) - 1)^{1/2} \cdot \exp(\sigma^2/2)} && (24)
\end{aligned}$$

yielding

$$\overline{Z} = \frac{\exp(\sigma\eta) - \exp(\sigma^2/2)}{(\exp(\sigma^2) - 1)^{1/2} \cdot \exp(\sigma^2/2)}. \tag{25}$$

Applying the lemma above to Eq. (25) for $y = \eta(\omega)$ yields the result.

As a corollary of this pointwise convergence, the distribution of $\overline{Z}$ converges to a standard normal distribution (as $\sigma \to 0^+$). Note, however, that convergence in distribution can also be proven for sums of log-normally distributed random variables, in contrast to pointwise convergence [4].

Supplementary Figures

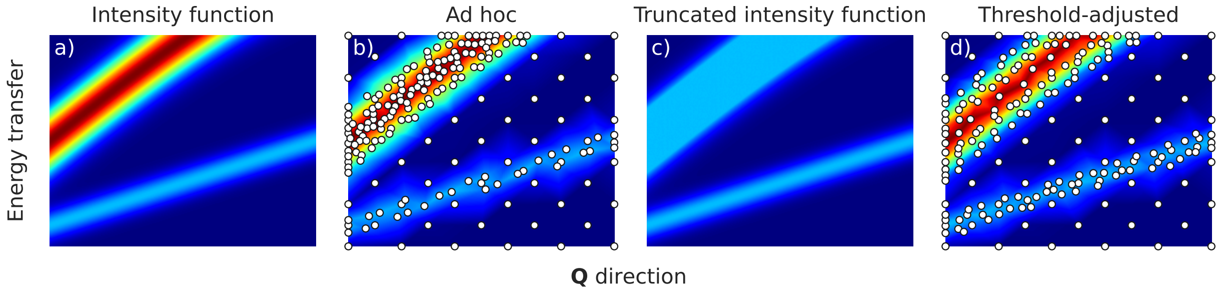

Supplementary Figure 1: Effect of intensity threshold. a) Intensity function i with two signal regions of different intensity magnitudes. b) An ad hoc use of corresponding intensity observations for conditioning the log-Gaussian process might place a large part of measurement points (dots) in the high intensity region. c) To solve this problem, the intensity observations are truncated to a threshold $\tau > 0$ such that the process can be thought of as fitted to $\min\{i(\mathbf{x}), \tau\}$. d) Using threshold-adjusted intensity observations yields measurement points that are more evenly distributed among the two different signal regions. Note that b) and d) contain the same number of 200 measurement points.

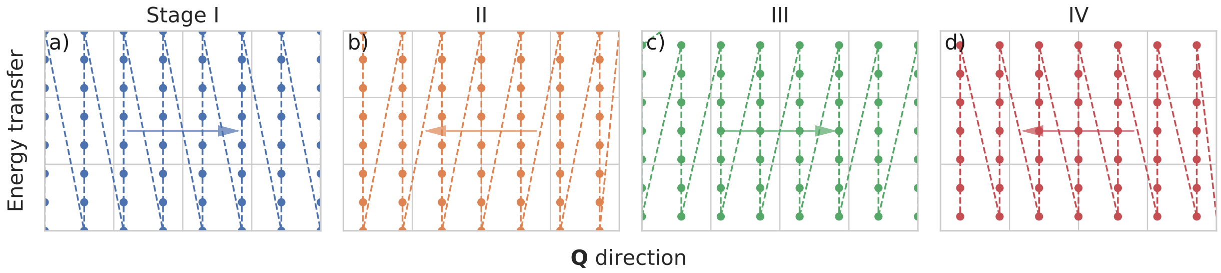

Supplementary Figure 2: Four stages I-IV of the grid approach. The arrows indicate the order of intensity observations (dots) in each stage (a-d).

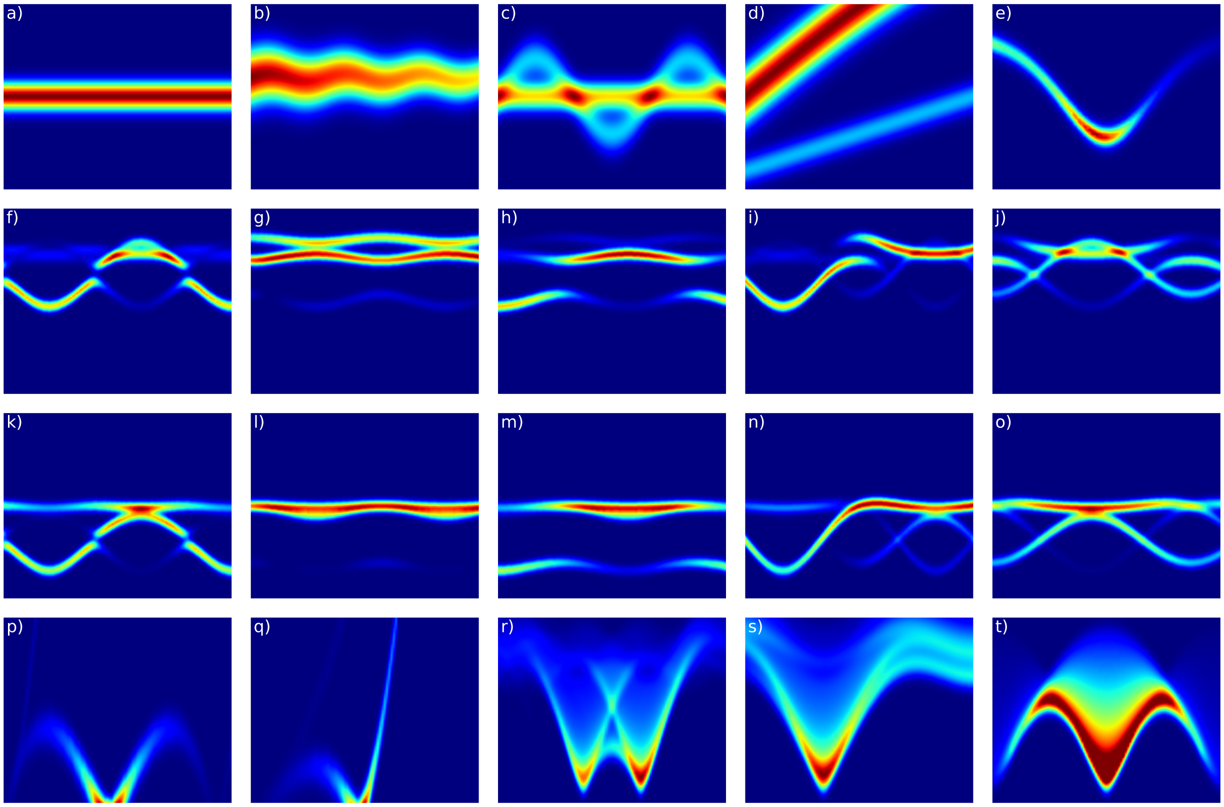

Supplementary Figure 3: Intensity functions of benchmark test cases. a),b) Crystal field excitations. c) Superposition of a crystal field excitation and a phonon. d) Two separate regions of signal with different intensity magnitudes. e) Transverse optical phonon in SnTe [5, 6]. f)-o) Spin wave spectrum of $Yb_2Ti_2O_7$ (see tutorial 20 of SpinW [7]). p),q) Spin waves in $ZnCr_2Se_4$ [8, 9]. r),s) Spin waves in FeP [10]. t) Spinon continuum in $SrCo_2V_2O_8$ [11].

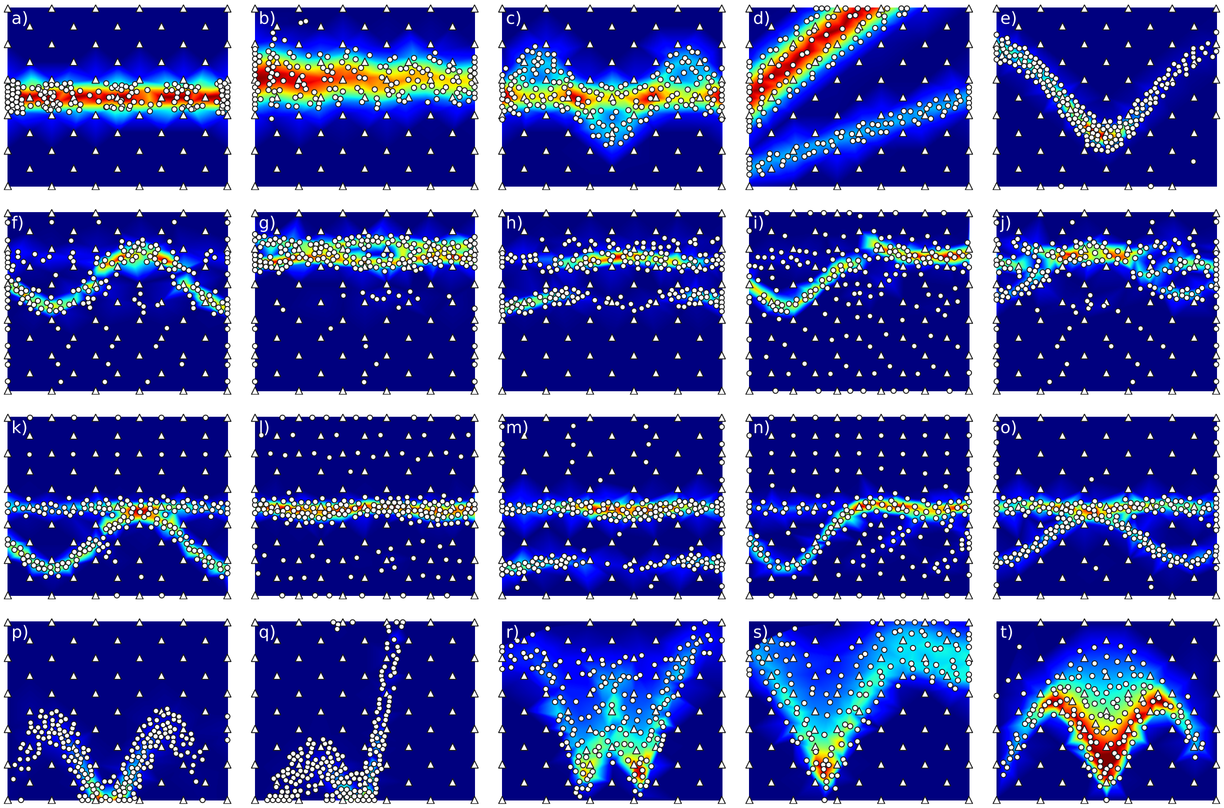

Supplementary Figure 4: Examples of particular experiments performed by our approach for each benchmark test case. a)-t) refer to intensity functions in Supplementary Fig. 3. Triangles represent the initialization grid and dots show locations of intensity observations autonomously placed by our approach. After initialization, measurement points are mainly placed in regions of signal for each test case.

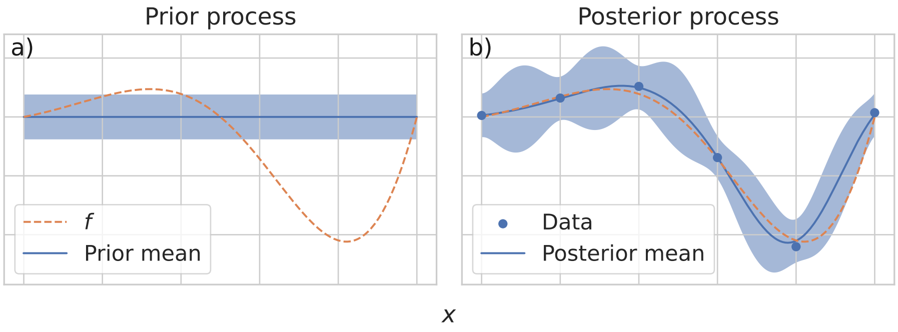

Supplementary Figure 5: Transition from a prior to a posterior process. The example function $f(x) = \sin(2\pi x)\exp(3x)$ defined on $[0, 1]$ is depicted by a dashed orange line. a) Prior process with its mean function (solid blue line) and its uncertainties (light blue area) as a 95% credible region between the 2.5% and 97.5% quantile. b) Posterior process with noisy data points of f (blue dots).

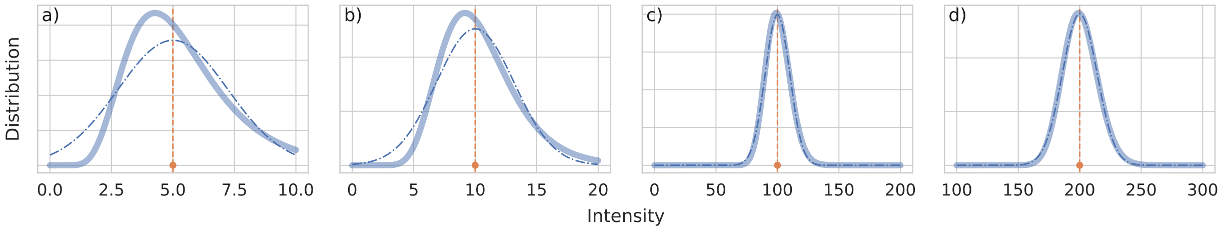

Supplementary Figure 6: Convergence of log-normal noise distribution. For increasing intensities $i(\mathbf{x}_j) \in \{5\ (a), 10\ (b), 100\ (c), 200\ (d)\}$ (orange dots and orange dashed lines), the noise random variable $\hat{I}(\mathbf{x}_j) \mid I(\mathbf{x}_j) = i(\mathbf{x}_j)$ (light blue stripe) converges in distribution to $I^+(\mathbf{x}_j) \mid I(\mathbf{x}_j) = i(\mathbf{x}_j)$ (dashed blue line), i. e., to a normal distribution.

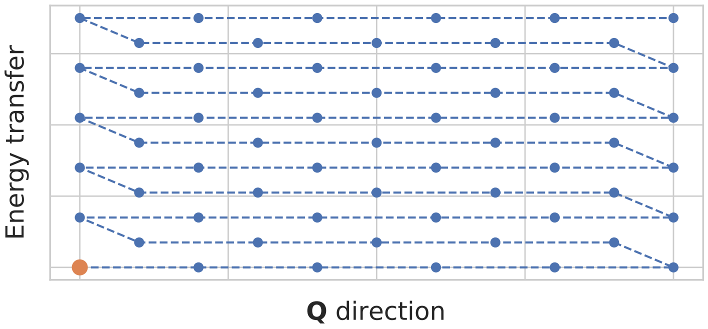

Supplementary Figure 7: Initial measurement locations arranged as a certain grid. Observations (blue dots) start at the bottom left corner (orange dot) and then continue row by row (dashed blue line).

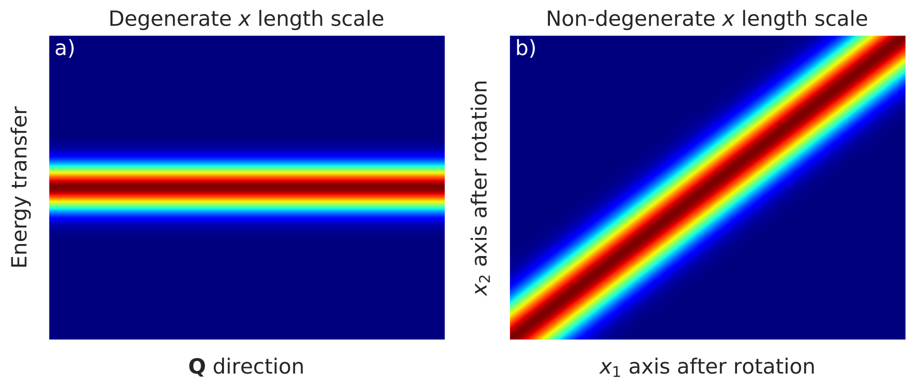

Supplementary Figure 8: Example of degenerate and non-degenerate length scale hyperparameters. a) Lower-dimensional intensity function yielding a degenerate length scale hyperparameter $\lambda_1 = \infty$. b) The same intensity function getting full-dimensional after a rotation of the coordinate system by 45°, thus allowing non-degenerate length scales $\lambda_k < \infty$.

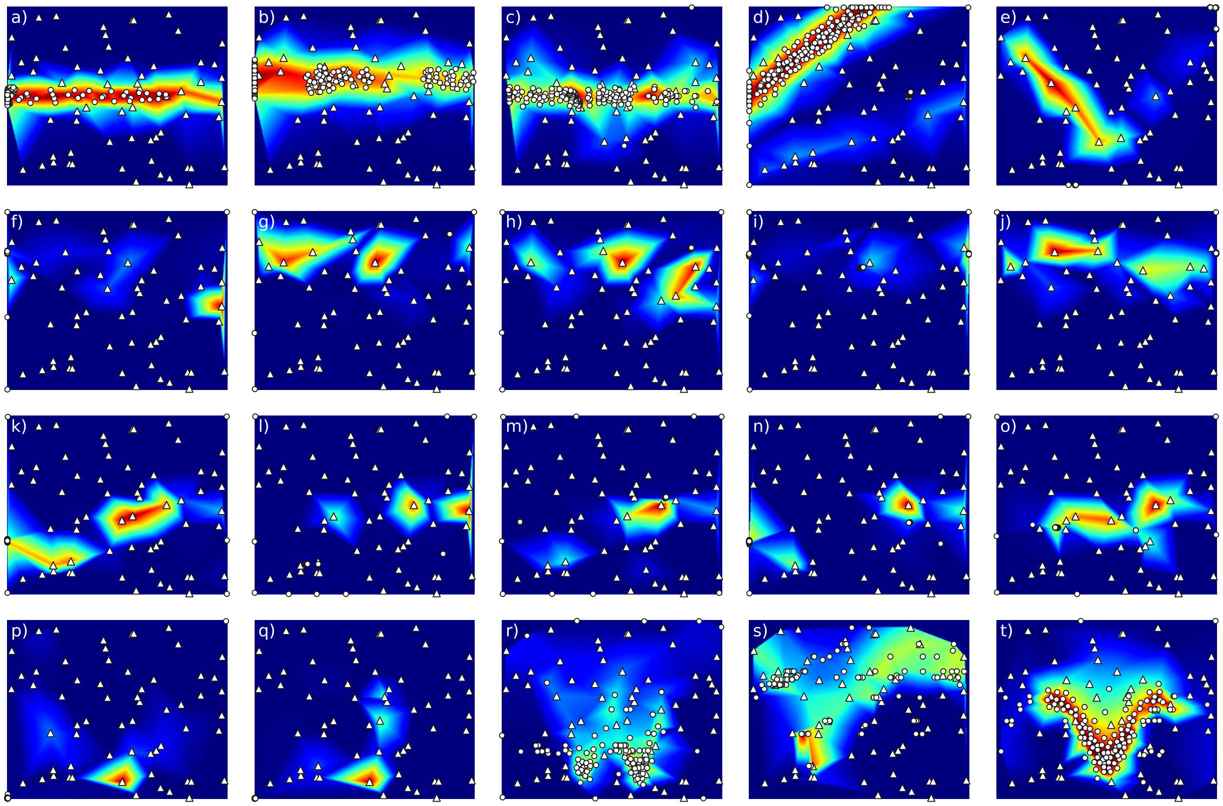

Supplementary Figure 9: Examples of particular experiments performed by gpCAM in the default setting for each benchmark test case. a)-t) refer to intensity functions in Supplementary Fig. 3. Triangles represent initial measurement locations and dots show those autonomously placed by gpCAM. In most test cases, the quality of experiments is rather poor.

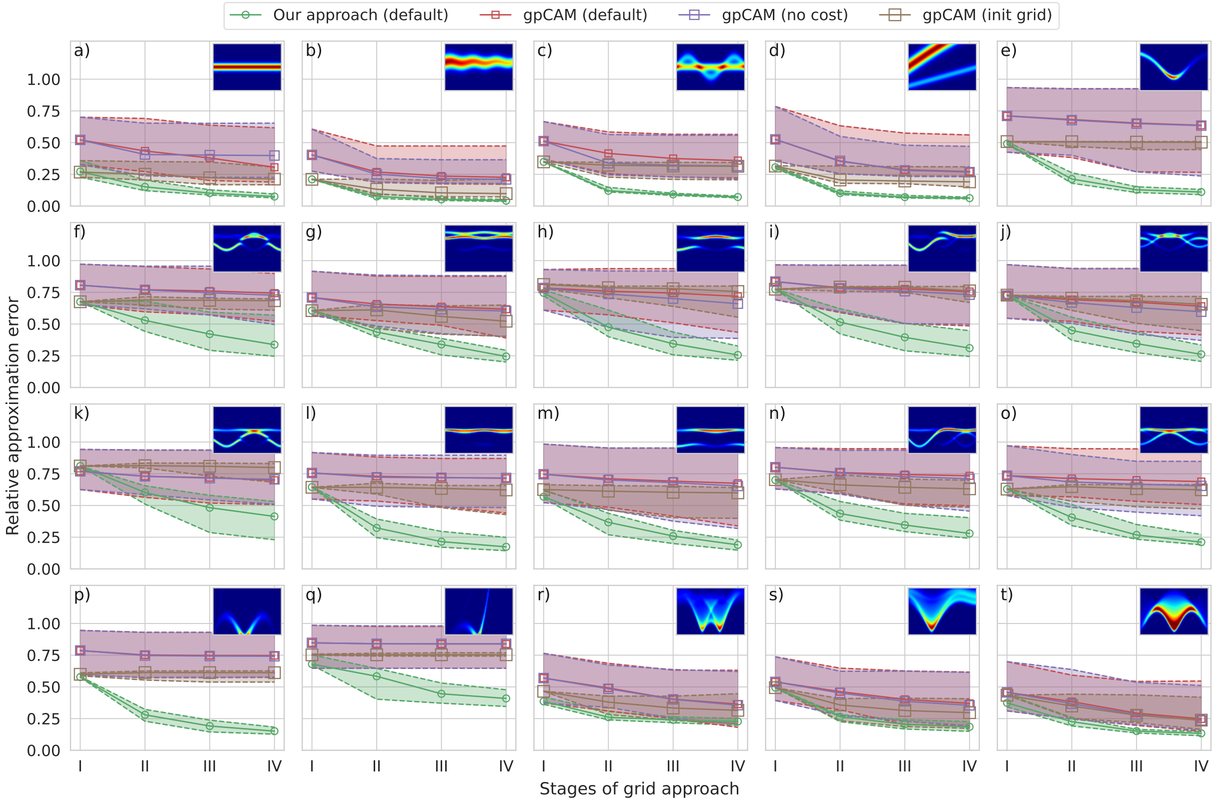

Supplementary Figure 10: Benchmark results of gpCAM. For each approach, every subfigure (a-t) plots the decay of a relative approximation error (Eq. (7) in the main text) between the target intensity function of the corresponding test case (top right corners) and a linear interpolation of collected intensity observations for four milestone values (symbols) which are determined by the four stages (I-IV) of the grid approach. The solid lines show medians of the resulting benefit values, whereas the light color areas with dashed boundaries indicate the range between their minimum and maximum to visualize their variability caused by stochastic elements. Our approach (green) performs significantly better than all gpCAM variants, both in terms of median average and variability of the results.

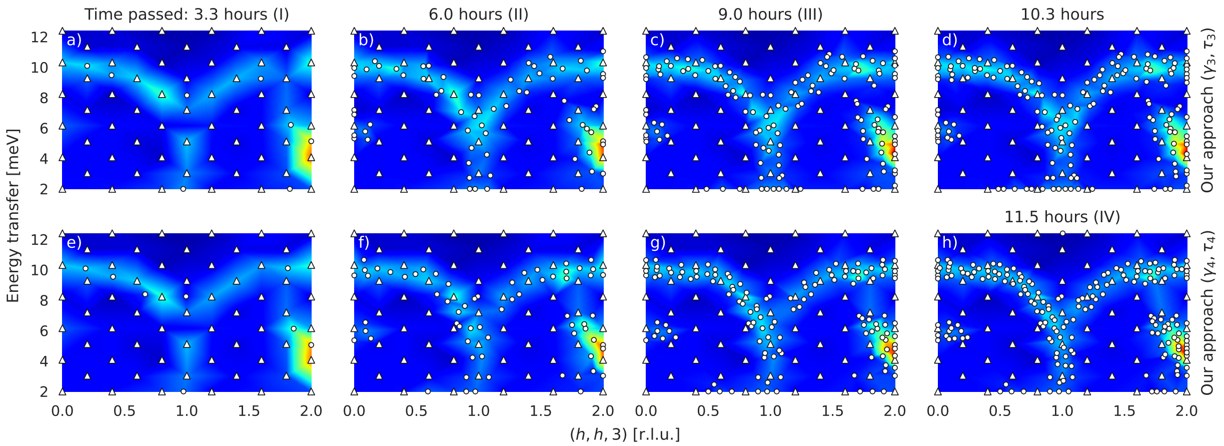

Supplementary Figure 11: Further results for scenario 1 in two additional settings of our approach. For the **Q** direction, we use relative lattice units (r.l.u.). The columns again indicate the four stages (I-IV) of the grid approach (see top row in Fig. 4). However, note that the last column of the top row is related to a different total experimental time since the corresponding experiment did not reach the final time of stage IV. Triangles again represent the initialization grid and dots show locations of intensity observations autonomously placed by our approach. The top row (a-d) corresponds to $\gamma_3 = 45$ and $\tau_3 = 90$ and the bottom row (e-h) to $\gamma_4 = \gamma_3$ and $\tau_4 = 130$.

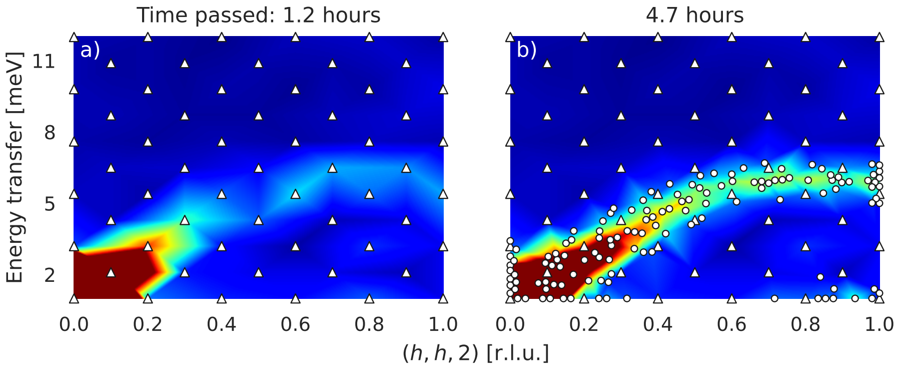

Supplementary Figure 12: Results for scenario 3. For the **Q** direction, we use relative lattice units (r.l.u.). a) Initial measurement points (triangles). b) Intensity observations (dots) autonomously placed after initialization.

Supplementary References

1. Schneidewind, A. & Čermák, P. PANDA: Cold three axes spectrometer. *Journal of large-scale research facilities* **1**, A12 (2015). DOI: 10.17815/jlsrf-1-35.
2. Stuhr, U. *et al.* The thermal triple-axis-spectrometer EIGER at the continuous spallation source SINQ. *Nucl. Instrum. Methods Phys. Res. A: Accel. Spectrom. Detect. Assoc. Equip.* **853**, 16–19 (2017). DOI: 10.1016/j.nima.2017.02.003.
3. Noack, M. M. *et al.* Gaussian processes for autonomous data acquisition at large-scale synchrotron and neutron facilities. *Nat. Rev. Phys.* **3**, 685–697 (2021). DOI: 10.1038/s42254-021-00345-y.
4. Dufresne, D. The log-normal approximation in financial and other computations. *Adv. Appl. Probab.* **36**, 747–773 (2004). DOI: 10.1239/aap/1093962232.
5. Heid, R. & Bohnen, K.-P. Linear response in a density-functional mixed-basis approach. *Phys. Rev. B* **60**, R3709 (1999). DOI: 10.1103/PhysRevB.60.R3709.
6. Li, Z. *et al.* Anomalous transverse optical phonons in SnTe and PbTe. *Phys. Rev. B* **105**, 014308 (2022). DOI: 10.1103/PhysRevB.105.014308.
7. Toth, S. & Lake, B. Linear spin wave theory for single-Q incommensurate magnetic structures. *J. Phys. Condens. Matter* **27**, 166002 (2015). DOI: 10.1088/0953-8984/27/16/166002.
8. Tymoshenko, Y. *et al.* Pseudo-Goldstone magnons in the frustrated $S = 3/2$ Heisenberg helimagnet $ZnCr_2Se_4$ with a pyrochlore magnetic sublattice. *Phys. Rev. X* **7**, 041049 (2017). DOI: 10.1103/PhysRevX.7.041049.
9. Inosov, D. S. *et al.* Magnetic field dependence of low-energy magnons, anisotropic heat conduction, and spontaneous relaxation of magnetic domains in the cubic helimagnet $ZnCr_2Se_4$. *Phys. Rev. B* **102**, 184431 (2020). DOI: 10.1103/PhysRevB.102.184431.
10. Sukhanov, A. *et al.* Frustration model and spin excitations in the helimagnet FeP. *Phys. Rev. B* **105**, 134424 (2022). DOI: 10.1103/PhysRevB.105.134424.
11. Bera, A. *et al.* Spinon confinement in a quasi-one-dimensional anisotropic Heisenberg magnet. *Phys. Rev. B* **96**, 054423 (2017). DOI: 10.1103/PhysRevB.96.054423.
