# Supplementary material for: Active learning-assisted neutron spectroscopy with log-Gaussian processes
Source: Nat Commun. 2023 Apr 19;14:2246. doi: 10.1038/s41467-023-37418-8 (PMC10115805; doi:10.1038/s41467-023-37418-8)
Supplement: Supplementary file 3 — Description of Additional Supplementary Files [file 41467_2023_37418_MOESM3_ESM.pdf]

File name: Supplementary Movie 1

Description: Results for scenario 1 of the neutron experiment in the default setting of our approach ($\gamma_0=58$ and $\ tau_0=94.5$ were automatically computed). It refers to row 2 in Fig. 4.

File name: Supplementary Movie 2

Description: Results for scenario 1 of the neutron experiment with $\gamma_1=\gamma_0$ and $\ tau_1=130$. It refers to row 3 in Fig. 4.

File name: Supplementary Movie 3

Description: Results for scenario 1 of the neutron experiment with $\gamma_2=30$ and $\tau_2=\ tau_0$. It refers to row 4 in Fig. 4

File name: Supplementary Movie 4

Description: Results for scenario 2 of the neutron experiment. It refers to Fig. 5.

File name: Supplementary Movie 5

Description: Results for scenario 3 of the neutron experiment. It refers to Supplementary Fig. 12.
